# An In Vitro Model for Characterization of Drug Permeability across the Tympanic Membrane

**DOI:** 10.3390/ph15091114

**Published:** 2022-09-07

**Authors:** Joachim G. S. Veit, Bhaskar Birru, Ruby Singh, Elizabeth M. Arrigali, Monica A. Serban

**Affiliations:** 1 Department of Biomedical and Pharmaceutical Sciences, University of Montana, Missoula, MT 59812, USA; 2 Montana Biotechnology Center (BIOTECH), University of Montana, Missoula, MT 59812, USA

**Keywords:** tympanic membrane, drug permeation, in vitro 3D tissue model, oto-therapeutics

## Abstract

Otic disorders, such as otitis media and hearing loss, affect a substantial portion of the global population. Despite this, oto-therapeutics, in particular those intended to treat hearing loss, have seen limited development and innovation. A significant factor to this is likely a result of the inherent costs and complexities of drug discovery and development. With in vitro 3D tissue models seeing increased utility for the rapid, high-throughput screening of drug candidates, it stands to reason that the field of otology could greatly benefit from such innovations. In this study, we propose and describe an in vitro 3D model, designed using a physiologically based approach, which we suggest can be used to estimate drug permeability across human tympanic membranes (TM). We characterize the permeability properties of several template drugs in this model under various growth and storage conditions. The availability of such cost-effective, rapid, high-throughput screening tools should allow for increased innovation and the discovery of novel drug candidates over the currently used animal models. In the context of this TM permeation model, it may promote the development of topical drugs and formulations that can non-invasively traverse the TM and provide tissue-targeted drug delivery as an alternative to systemic treatment, an objective which has seen limited study until present.

## 1. Introduction

Disorders of the ear, including otitis media, noise-induced hearing loss, and other otological ailments, affect hundreds of millions of adults and children worldwide [1,2,3]. Despite this, there are currently no approved medications treating hearing loss [4], and disorders like otitis media are typically treated systemically or using invasive techniques such as tympanostomy tubes or tympanic membrane (TM)-perforating injections [5,6].

Therapeutics that would allow for non-invasive topical drug delivery across the TM would result in significant medical and economic advantages over the current methods. These would allow for tissue-targeted delivery and reduce unnecessary systemic exposure and adverse effects, while reducing the need for invasive, costly, and risky medical procedures. Unfortunately, this approach is challenging because the TM is highly impermeable to most compounds [5,7]. This likely limits significant study into novel permeable therapeutics; however, previous studies have shown permeability improvements are possible with formulation changes [8,9]. Current methods of screening TM permeability primarily rely on expensive, labor-intensive, and low-throughput animal models such chinchillas [8,9], posing a significant barrier to rapid drug development. Additionally, animal tissues do not accurately represent human anatomy and physiology; therefore, an in vitro model based on human tissues could potentially be more advantageous. Relying on animal models alone would likely result in otherwise effective drug candidates remaining undiscovered or untested. Improved tools for studying and developing oto-therapeutics would allow for the high-throughput, low-cost, accelerated screening of novel drug candidates leading to increased discovery and improved patient outcomes. Additionally, therapeutics that would allow for non-invasive topical drug delivery across the TM would result in significant medical and economic advantages over the current methods for the significant portion of the global population affected by these ailments.

**Figure 1:**
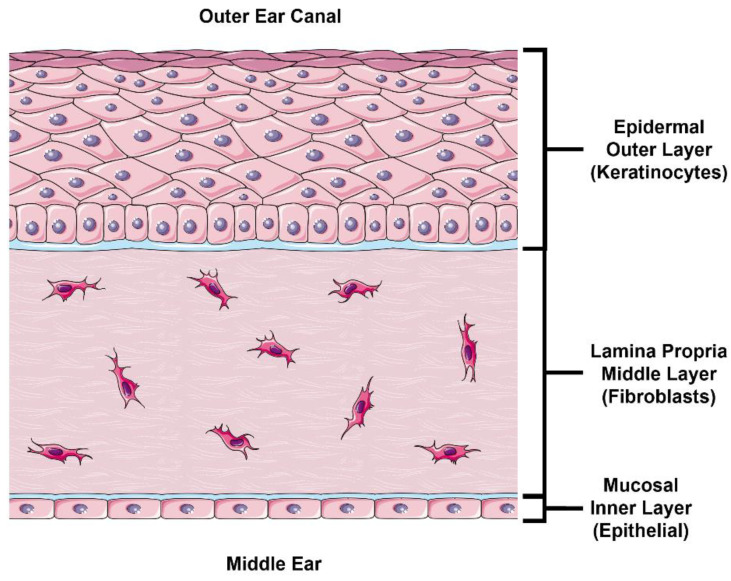
**Illustration of TM Cross Section.** The outer layer of the TM consists of a stratified epidermis skin layer consisting primarily of keratinocytes. The lamina propria (middle layer) consists of fibroblasts embedded in connective tissue (primarily collagen and elastin). The inner (middle ear) layer of the TM consists of a thin mucosal epithelial layer. *TM, tympanic membrane. This figure was drawn using modified artwork from Servier Medical Art. Servier Medical Art by Servier is licensed under a Creative Commons Attribution 3.0 Unported License (https://creativecommons.org/licenses/by/3.0/,* *accessed on 25 June 2022).*

In this study, we follow a physiologically based approach at developing an in vitro model to estimate TM drug permeability. The external layer of the TM is continuous with the skin in the external acoustic meatus and comprised of stratified epidermal keratinocytes (Figure 1, top). The outermost portion of this epidermal layer is composed of a lipid- and protein-rich stratum corneum (SC) formed by terminally differentiated keratinocytes known as corneocytes. This layer is likely the most significant barrier to drug permeation [7], and most drugs undergo paracellular permeation in this layer [10], which requires diffusion across the lipid-rich intercellular “mortar” of the SC. Below the SC exists the stratum granulosum, followed by the stratum spinosum, which both contain intercellular tight junctions that control and limit paracellular drug permeation [11]. This obstruction of paracellular permeation necessarily results in a relative increase in the role of transcellular permeation, which requires diffusion or transport across cell membranes and cytoplasm. The lower layer of the epidermis consists of proliferating basal keratinocytes that replenish the superficial keratinocyte layers. Underneath the epidermal layer lies the lamina propria (Figure 1, middle), composed of fibroblasts in collagen-rich porous connective tissue. Intuitively, this layer is unlikely to hinder any significant drug permeation in steady-state conditions, which we confirm in this study. Finally, the inner mucosal layer of the TM comprises a thin epithelial cell layer (Figure 1, bottom). Although this layer does express tight junction proteins [12], it does not have an SC like the epidermis and is not expected to be as nearly as impermeable as the epidermal layer [5].

Herein, we propose that this in vitro model may function as a useful representation of human TM permeability properties for the purposes of the rapid, cost-effective, high-throughput screening of oto-therapeutics. We introduce our physiologically based rational-to-TM model design, histologically characterize our model in several growth and storage conditions, and determine the drug permeability of four representative small-molecule drugs across our model in various growth and storage conditions.

## 2. Results

### 2.1. Modeling the TM Outer Layer

**Figure 2:**
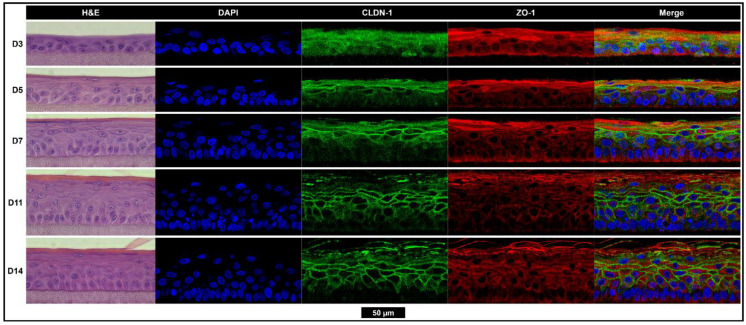
Tissues are morphologically fully developed after 11 days of growth with the strong expression of tight junction proteins CLDN-1 and ZO-1. The hematoxylin and eosin (left), nuclear (blue), CLDN-1 (green), and ZO-1 (red) staining of epidermis-like stratified keratinocyte tissues grown while exposed to the air–liquid interface for 3, 5, 7, 11, or 14 days. All images scaled to match the black scale bar (50 µm). *CLDN-1, claudin-1; D, day; DAPI, 4′,6-diamidino-2-phenylindole; H&E, hematoxylin and eosin; ZO-1, zona occludens-1.*

The outer layer of the human TM consists of a thin layer of skin consisting of stratified keratinocytes. Like the skin, this layer contains tight junctions and a lipid-rich outer corneal layer of terminally differentiated cornified keratinocytes (corneocytes) that act as robust physiological barriers. Primary keratinocytes were grown at an air–liquid interface (ALI) for 3 to 14 days to monitor stratification, differentiation, and tight junction protein expression changes and to determine when the tissues have fully formed (Figure 2). Morphologically, the tissues were fully developed by day 11 at ALI. Staining for tight junction protein claudin-1 can be seen throughout the middle layers (stratum spinosum) and zona occludens 1 (ZO-1), with the staining strongest in the outermost viable layer (stratum granulosum).

**Figure 3:**
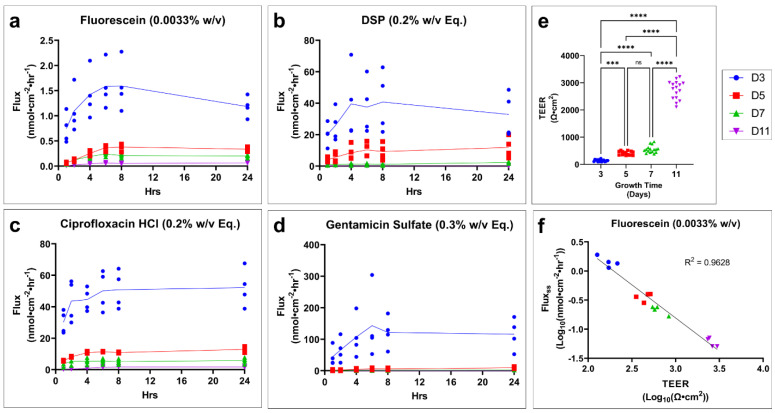
**Tissue growth time has a significant effect on TM model drug permeation and TEER.** Drug flux of profile over time for (**a**) fluorescein, (**b**) DSP, (**c**) ciprofloxacin HCl, and (**d**) gentamicin sulfate grown for 3, 5, 6, or 11 days. *n* = 3–4. (**e**) Effect of growth time on the TEER of tissues. *n* = 15–16. One-way ANOVA with Tukey’s correction for multiple comparisons; *** *p* < 0.001, **** *p* < 0.0001 (**f**) Example of correlation between fluorescein flux at steady state (flux_ss_) and TEER after log transformation. *n* = 4 (16 total). Legend applies to all panels. *DSP, dexamethasone sodium phosphate; Eq, molar equivalent of base compound; TEER, transepithelial electrical resistance; TM, tympanic membrane; w/v, weight by volume; ns, not significant.*

During the maturation process of the tissues, a dramatic decrease in fluorescein, DSP, ciprofloxacin HCl, and gentamicin sulfate flux (Figure 3a–d, respectively) is seen as tissue growth time increases. Transepithelial electrical resistance (TEER), which is an indirect measure of barrier function based on ion permeability, steadily increases with growth time (Figure 3e). The log transformation of the TEER and flux value highlights a strong inverse relationship between TEER and drug flux at steady state (Figure 3f, Supplemental Figure S1).

**Figure 4:**
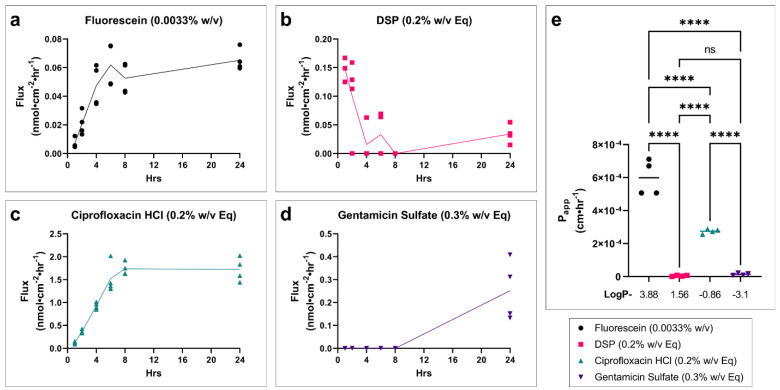
**Drug permeability profiles and relative drug permeability for fully developed in vitro TM models.** Drug flux of profile over time for (**a**) fluorescein, (**b**) DSP, (**c**) ciprofloxacin HCl, and (**d**) gentamicin sulfate in a fully developed TM model grown for 11 days at the air–liquid interface. *n* = 4. (**e**) Apparent permeability coefficient (P_app_) at approximate steady-state conditions (average of 6–24 h timepoints), for each drug in the fully developed in vitro TM model. The log P of each drug is shown below *x*-axis. *n* = 4; one-way ANOVA with Tukey’s correction for multiple comparisons; **** *p* < 0.0001. Legend applies to all panels. *DSP, dexamethasone sodium phosphate; Eq, molar equivalent of base compound; TEER, transepithelial electrical resistance; P_app_, apparent permeability coefficient; w/v, weight by volume; ns, not significant.*

The flux over time for each drug across fully developed tissues is shown. Fluorescein (Figure 4a) and ciprofloxacin (Figure 4c) show an expected slow increase in flux until steady state is achieved. DSP (Figure 4b) shows an initial burst in flux with a sharp decline to an approximate steady state. Gentamicin sulfate (Figure 4d) had such low permeability that it remained below the limit of detection until the longer collection interval between 8 and 24 h. Apparent permeability coefficient (P_app_) corrects for the concentration gradient across the tissue using steady-state flux in order to better compare drugs to each other. Fluorescein shows the highest permeability, followed by ciprofloxacin (Figure 4e). DSP and gentamicin sulfate have the lowest permeability and are not significantly different from each other.

### 2.2. Influence of TM Middle Layer Model on Drug Permeability

**Figure 5:**
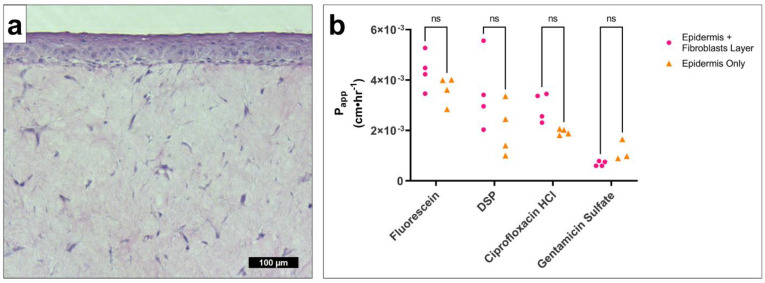
**Fibroblast connective tissue layer has no significant effect on drug permeability in the in vitro TM model.** (**a**) Representative micrograph of hematoxylin-and-eosin-stained epidermis with an underlying connective tissue layer with embedded fibroblasts. (**b**) P_app_ at approximate steady-state conditions (average of 6–24 h timepoints), for fluorescein (0.0033% *w/v*), DSP (0.2% *w/v* Eq), ciprofloxacin HCl (0.2% *w/v* Eq), and gentamicin sulfate (0.3% *w/v* Eq) in a TM model containing epidermis with connective tissue/fibroblasts (pink circle) or epidermis only (orange triangle). Underdeveloped tissues were tested (5 days growth) to increase resolution and avoid detection limits artificially reducing of any differences. *n* = 3–4. Multiple t-tests with Holm–Šídák correction for multiple comparisons. *DSP, dexamethasone sodium phosphate; Eq, molar equivalent of base compound; ns, not significant; P_app_, apparent permeability coefficient; TM, tympanic membrane; w/v, weight by volume.*

Human fibroblasts embedded in a collagen matrix were used to model the lamina propria of the TM. Epidermal keratinocyte tissues were grown with (Figure 5a) or without this underlying fibroblast connective tissue layer, and their permeabilities were compared (Figure 5b, Supplemental Figure S2). Day 5 tissues were selected, rather than fully developed tissue, to prevent drug detection limits masking the effect of the lamina propria on drug permeability. We found no significant difference in P_app_ in any of the drugs tested.

### 2.3. Effect of Freeze/Thaw Cycles on TM Model Barrier Properties and Permeability

**Figure 6:**
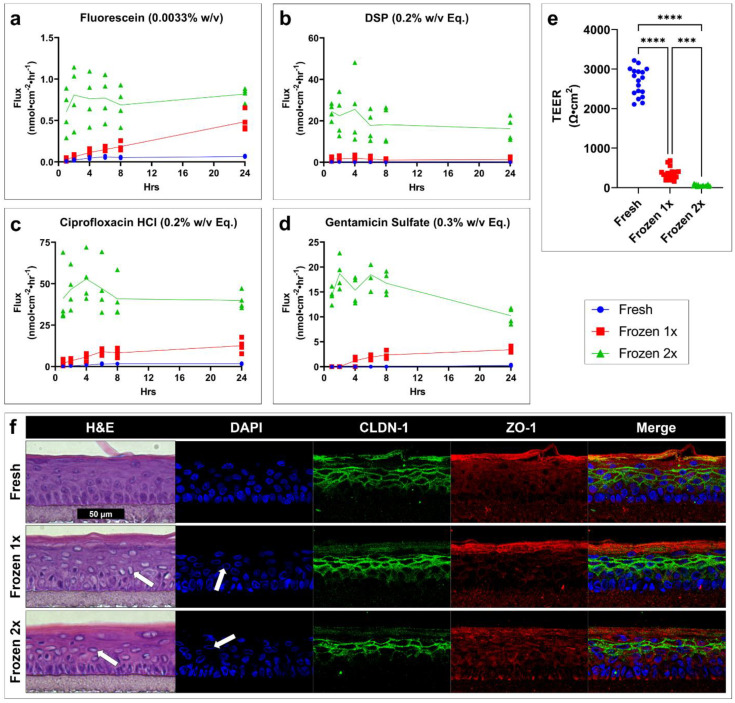
Freeze–thaw cycles greatly increase drug permeability, decrease TEER, and disrupt cell and tissue morphology in an in vitro TM model. Drug flux of profile over time for (**a**) fluorescein, (**b**) DSP, (**c**) ciprofloxacin HCl, and (**d**) gentamicin sulfate in a fully developed TM model tested when fresh (blue circle) or having undergone one (red square) or two (green triangle) freeze–thaw cycles. *n* = 4. (**e**) TEER of a fully developed TM model tested when fresh (blue) or having undergone one (red) or two (green) freeze–thaw cycles. *n* = 18; one-way ANOVA with Dunnett’s correction for multiple comparisons; *** *p* < 0.001, **** *p* < 0.0001. (**f**) Representative micrographs of hematoxylin-and-eosin-, DAPI-, CLDN-1-, and ZO-1-stained tissues showing the morphological effects of freeze–thaw cycles. White arrows highlight examples of cellular and nuclear damage. All images scaled to match the black scale bar (50 µm). Legend applies to panels (**a**–**e**). *CLDN-1, claudin-1; DAPI, 4′,6-diamidino-2-phenylindole; DSP, dexamethasone sodium phosphate; Eq, molar equivalent of base compound; H&E, hematoxylin and eosin; TEER, transepithelial electrical resistance; w/v, weight by volume; ZO-1, zona occludens-1.*

The flux over time for each drug is presented (Figure 6a–d). Fresh, viable tissues (blue circle) show the lowest permeability, while one (red square) or two (green triangle) freeze–thaw cycles result in respective increases in drug permeability. TEER (Figure 6e) significantly decreases as a result of freeze–thaw cycles. Histology reveals structural damage to cells and disrupted nuclear staining (Figure 6f, white arrows). CLDN-1 staining is minimally affected, with only a slight reduction in lower layer staining in the two freeze–thaw cycle tissues. ZO-1 staining is not noticeably changed.

## 3. Discussion

This study introduces the possibility that a physiologically based in vitro model of the TM can be used to adequately estimate drug permeability across the TM in a cost-effective high-throughput manner relative to current options. Our approach presumes that TM permeability properties can be adequately mimicked by investigating the contributions of individual TM layers. Due to its homologous composition and continuity with the skin, the outer layer of the TM can be mimicked by an in vitro reconstructed epidermis, which contributes the most significant barrier to drug permeation. When we investigated the role of a fibroblast-embedded connective-tissue layer relative to the epidermal layer, we confirmed the lamina propria has no significant effect on drug permeability. In addition to expectations that the inner mucosal layer of the middle ear is not as impermeable as the rest of the TM [5], our own study into the permeation of a mucosal epithelial layer intended to mimic the outer layer of the RWM (which is equivalent to and continuous with the middle ear epithelium and inner layer of the TM) confirms this expectation and showed a minimum of 80, and up to 6000-fold higher, drug permeability than our epidermal TM models [13].

Using this information, we predicted that the drug permeation properties of a human TM could be approximated using an in vitro model of a fully developed human epidermis. We began this exploration by determining the effect of growth time on tissue morphology, tight junction formation, drug permeation, and TEER. The drugs used for permeation testing were selected to have a wide range of partition coefficients to allow for the elucidation of the contribution this physiochemical property to model permeability. Additionally, previous use in human TM permeation studies [6,14], general applicability as oto-therapeutics, and ease of detection were also considered. Our results indicated that 11 days at ALI was sufficient to be considered fully developed and allowed for the formation of the SC and tight junctions thought to be the major contributors to drug permeation. The collection permeation data at shorter growth times can be used to retrospectively adjust this model as it becomes validated with currently accepted animal models or with data from human TM tissues.

By using P_app_ to investigate the relative permeability of the tested drugs, we see a trend of decreasing permeability with decreasing partition coefficient (log P) (fluorescein log P = 3.88, ciprofloxacin HCl log P = −0.86, gentamicin sulfate log P = −3.1) [15,16,17], with exception of DSP (log P = 1.56) [18], which appears to be an outlier to this trend. This discrepancy and the abnormal inverted flux trend of DSP seen in our data may be explained by the rapid hydrolysis of DSP (pro-drug) into dexamethasone (active form) by phosphatases [19,20]. If a majority of DSP is converted to the more lipophillic dexamethasone (log P = 1.68) [21], which was not quantified in our analysis, the permeation of the less lipophilic DSP would not represent the true overall permeability of the drug, and much of the permeated drug would not be accounted for in the analysis. Since we would expect a more lipophilic drug to show improved permeability through the SC and across cell membrane lipid-bilayers, it stands to reason that dexamethasone would permeate more freely than DSP [22]. This pattern in DSP flux is not observed in undeveloped or freezing-damaged tissues. This is likely due to the underdeveloped or damaged SC and epidermis allowing the increased permeation of non-hydrolyzed DSP. Although it was not within the scope of our study, this conversion of DSP to dexamethasone and the relative permeability of each would warrant further investigation and elucidation.

It is well understood that freeze–thaw cycles will cause mechanical damage to biological tissues. Our study found a direct relationship between freeze–thaw cycles resulting in increased drug permeability and decreased TEER. This effect of freezing on TEER, an analog for barrier integrity, matches the previously reported effect in human cadaveric TM [6]. Morphologically, we see clear evidence of tissue damage, most apparently internal cellular damage, as evidenced by the deterioration of the nucleus. We saw significantly fewer effects on tight junction protein organization, which suggests that the increase in drug permeability resulted from increased transcellular, rather than paracellular, permeation.

The physiological relevance of this model seems to be additionally supported by a published study on the assessment of ciprofloxacin permeation in fresh in situ human cadaveric TMs, [6] as well as our own in situ frozen human cadaveric TM studies [14] (Supplemental Figure S3). Both in situ TM studies also found that the permeability of the TM barrier is compromised by freeze/thawing, in line with our in vitro TM model findings. Although it was not investigated in this work, the physiological relevance of this model may also permit additional research utility beyond drug permeation studies. Further studies could build upon this model to develop versions that mimic common TM disease states by utilizing relevant patient donor cells rather than healthy cells. Additionally, this or similar models may also allow researchers to more easily study drug-induced tissue damage and the effects of drug–drug interactions, which are known to be capable of affecting TM integrity [23].

Overall, this study underlines the feasibility of using deconstructed, yet physiologically relevant, in vitro model systems for the development and screening of topical oto-therapeutics, and potentially for personalized therapies for rare diseases or to model various ear-specific disease states. Additionally, such models can allow the independent evaluation of drug permeation properties and drug delivery systems to provide alternative methods for optimizing topical oto-therapeutics safety and efficacy.

## 4. Materials and Methods

### 4.1. Tissue Culture

Human primary neonatal epidermal keratinocytes (Gibco, C-001-5C, Burlington, MA, USA) were proliferated in Epilife complete media (Epilife (Gibco, 15140-122, Burlington, MA, USA) with HKGS (Gibco, S0015, Burlington, MA, USA) and 0.5% penicillin/streptomycin (Gibco, 15140-122, Burlington, MA, USA) as previously described [24,25]. Keratinocytes were then seeded into 12 mm cell culture inserts (Millipore, PIHP01250, Burlington, MA, USA) (1.5 × 10^5^ cells/insert) and placed into a 6-well plate containing Epilife complete media supplemented with 1.44 mM calcium chloride and incubated for overnight in a 37 °C and 5% CO_2_ humidified incubator. The next day, the media in the wells was replaced with 1.5 mL Epilife complete supplemented with 1.44 mM calcium chloride, 91.4 µg·mL^−1^ 2-phospho-L-ascorbic acid trisodium salt, and 10 ng·mL^−1^ human keratinocyte growth factor (Sigma, K1757, St. Louis, MO, USA), which was replaced every 2 days for the remainder of the tissue growth. An air–liquid interface was established by drying the surface of the tissue, which was kept dry for the remainder of the tissue growth.

### 4.2. Tissue Freeze/Thaw Cycles

To test the effects of freeze–thaw cycles on tissue properties, the media was removed and the tissue plate was sealed with plastic wrap. The tissues were then placed into a room-temperature closed-cell polystyrene foam cooler, which was placed at −20 °C. After 24 h, the tissues were removed from the cooler and allowed to warm to room temperature for approximately 30 min before either testing or returning them to the cooler, then freezer for a second freeze–thaw cycle.

Dual-layer tissues containing an epidermal layer and an underlying collagen-embedded fibroblast layer were purchased at day 0 of ALI from Mattek Corporation (EFT-400-D0) and grown per manufacturer’s instructions until day 5.

### 4.3. Histology, Immuno-Labeling, and Imaging of Tissues

Tissues were fixed overnight (4% formaldehyde + 1% acetic acid in phosphate buffered saline (PBS)) then processed and paraffin-embedded using a Leica ASP300S tissue processor. Starting at 1.5 mm from the edge of the tissue, 6 µm sections were taken and mounted onto glass slides. The slides were either stained with hematoxylin and eosin (H&E) or deparaffinized and rehydrated for immuno-labeling in a Leica Autostainer XL. A Leica DMI3000B inverted microscope with a Leica DFC450C camera was used to image the H&E stained tissues.

For immuno-labeling, deparaffinized slides were placed in a double boiler containing 2.2 g·L^−1^ citric acid (pH 6) for 20 min for antigen retrieval. Slides were rinsed in PBS, then blocked for 1 h in a buffer containing 0.02% (*v/v*) triton X-100, 0.1 M glycine, and 1% (*w/v*) bovine serum albumin (BSA) in PBS. Primary antibodies diluted in PBT buffer (0.2% BSA and 0.02% triton X-100 in PBS) were incubated overnight at 4 °C in a humidity chamber. Slides were then washed 3 × 3 min in a PBT buffer with gentle orbital shaking. Secondary antibodies in a PBT buffer were incubated at room temperature for 1 h in a humidity chamber. Slides were washed 3 × 3 min in PBT buffer, then incubated with DAPI in PBT buffer for 10 min, followed by another 3 × 5 min wash in PBS. Mowiol + DABCO mounting media (10% *w/v* Mowiol 4-88, 25% *w/v* glycerol, 0.1 M Tris (pH 8.5), and 2.5% *w/v* 1,4-diazobicyclo-[2.2.2]-octane) was used to mount coverslips. Slides were immediately imaged on a Leica Stellaris 5 confocal microscope.

Primary antibodies goat anti-human ZO-1 (Invitrogen, PA5-19090, Waltham, MA, USA) and rabbit anti-human CLDN-1 (Invitrogen, 51-9000, Waltham, MA, USA) were diluted 1:200. Secondary antibody donkey anti-goat 647 (Invitrogen, A32849, Waltham, MA, USA) was diluted 1:500, and donkey anti-rabbit 555 (Invitrogen, A32794, Waltham, MA, USA) was diluted 1:1000. DAPI (Thermo Scientific, 62248, Waltham, MA, USA) was diluted 1:1000.

### 4.4. Permeation Devices

A custom permeation device (Supplemental Figure S4) was used for permeation testing and transepithelial electrical resistance (TEER) measurements to eliminate leakage between the interface of the cell culture insert and the tissue layers. The permeation devices were modeled in Fusion360 software (Autodesk, San Rafael, CA, USA), then exported to PreForm software for printing on a Form 3 (Formlabs, Somerville, MA, USA) photopolymer 3D printer using Clear V4 resin. The device was designed to contain a standard 24-well plate cell culture insert and fit within a standard 12-well cell culture plate acting as the receiver chamber. The tissue-containing insert is placed into the base unit, then the top unit, which contains a 6 mm inner diameter “donor” chamber, is placed over the tissue and secured in place with 3 stainless steel M1.6 bolts threaded into nuts that had been press-fit into the base, to form a seal separating the donor and receiver chambers. A secondary channel leading to the receiver chamber allows for a TEER electrode to be placed while the second electrode is placed into the donor chamber.

### 4.5. Transepithelial Electrical Resistance

TEER was evaluated with a Millicell ERS-2 voltohmmeter (Millipore, MERS00002). This was performed prior to permeation testing to ensure the device was properly mounted and that the tissue was undamaged before testing was performed. Tissues in devices were placed into a 12-well plate containing 0.8 mL PBS, and an additional 0.2 mL PBS was placed into the donor chamber. After checking for any trapped air, one electrode was placed into the donor chamber, and the other was placed into the outer electrode channel (Supplemental Figure S4a,b). The TEER reading was allowed to stabilize and the value was recorded. TEER was also recorded using an insert without a tissue to act as a blank. All TEER values were blank-adjusted, then multiplied by the permeable surface area (0.2827 cm^2^) and reported as impedance·area (Ω·cm^2^).

### 4.6. Drug Preparation

Fluorescein (Fluka Analytical, 32615-25G-R) was prepared at 10 mM (3.32 mg/mL) in nanopure water. Then, 1 M sodium hydroxide was slowly added until it fully dissolved, then the pH was brought back to 8.5 ± 0.5 with 1 M hydrochloric acid (HCl). This 100× solution was stored at −20 °C until use and diluted to 100 µm with PBS prior to testing. Dexamethasone 21-phosphate disodium (DSP) (Alfa Aesar, J64083, Haverhill, MA, USA) was prepared at 2.63 mg·mL^−1^ (equivalent to 0.2% *w/v* dexamethasone) in PBS. Ciprofloxacin HCl monohydrate (Alfa Aesar, J61970, Haverhill, MA, USA) was prepared at 2.33 mg·mL^−1^ (equivalent to 0.2% *w/v* ciprofloxacin) in PBS, and the pH was reduced to 3.5 ± 0.5 with 1 M HCl to solubilize. Gentamicin sulfate (Acros Organics, 61398-010, Waltham, MA, USA) was prepared at 3.62 mg·mL^−1^ (equivalent to 0.3% *w/v* gentamicin) in PBS. The concentration of each drug (except fluorescein, which was chosen arbitrarily) was selected to match the currently available clinical formulations for each.

### 4.7. Drug Permeation Testing

Following TEER measurement, tissues in devices were placed into 12-well plates containing 0.6 mL PBS taking care not to trap any air bubbles. A total of 0.2 mL of each drug solution was placed into the donor chamber; the plates were covered, then placed into a 37 °C and 5% CO_2_ humidified incubator. At each timepoint (1, 2, 4, 6, 8, and 24 h) the tissues were moved into a new well containing fresh PBS. To allow for the accurate determination of steady-state (SS) flux and the apparent permeability coefficient (P_app_), the drug concentration in the donor chamber must remain constant. Therefore, at each timepoint, 100 µL of the donor solution was removed and replaced with fresh drug. We also confirmed that the receiver solution concentration never exceeded > 10% of the donor concentration to maintain the sink condition assumption [26]. The receiver solution from the completed timepoint is then collected and stored at −20 °C in a light-proof container until ready for analysis. A noticeable decrease in the drug solution level of the donor chamber at any point in the study indicated a leak in the device seal, and the sample was excluded from the study.

Fluorescein permeation was determined using a Cytation5 (Biotek) microplate reader (ex. 490 nm; em. 515 nm) against a standard curve. The other drugs were analyzed by reverse-phase UV–Vis high-performance liquid chromatography (HPLC).

### 4.8. High-Performance Liquid Chromatography

Samples and standards were filtered using 0.22 µm PVDF syringe filters prior to HPLC using an Agilent 1260 Infinity II system (Agilent Technologies Inc., Santa Clara, CA, USA). All reagents were HPLC-grade, and buffers were freshly made and filtered at 0.1 µm prior to use.

DSP: 50 µL injection volume into an isocratic mobile phase containing 75% 0.01 M potassium phosphate buffer (pH 7.58) and 25% acetonitrile at 1.5 mL·min^−1^. The column was a Gemini 3 µm 100 × 4.6 mm reverse-phase C_18_ column (Phenomenex, 00D-4439-E0, Torrance, CA, USA) set at 25 °C and protected by a SecurityGuard C_18_ 4 × 3.0 mm cartridge (Phenomenex, AJ0-7597, Torrance, CA, USA). A retention time of DSP at 1.9 min was used to quantify the area under the curve (AUC) of absorbance at 240 nm.

Ciprofloxacin: 50 µL injection volume into an isocratic mobile phase containing 80% 0.02 M potassium phosphate buffer (pH to 2.7 with orthophosphoric acid) and 20% acetonitrile at 1.0 mL·min^−1^. The column was a Gemini 5 µm 150 × 3 mm reverse-phase C_18_ column (Phenomenex, 00F-4435-Y0, Torrance, CA, USA) set at 25 °C and protected by a SecurityGuard C_18_ 4 × 2.0 mm cartridge (Phenomenex, AJ0-7596, Torrance, CA, USA). A retention time of ciprofloxacin at 1.75 min was used to quantify the AUC of absorbance at 277 nm.

Gentamicin sulfate: Analysis was performed using a modification of a previously described method [27,28]. The derivation reagent was prepared by dissolving 200 mg phthalaldehyde (TCI, P0280-100G, Tokyo, Japan) into a solution of 19 mL 0.4 M boric acid (pH 10.4), 1 mL methanol, and 0.4 mL thioglycolic acid (Fisherbrand AC125430010, Loughborough, UK), then the pH was modified to 10.4 with 8 N potassium hydroxide before filtering at 0.22 µm. The derivation was then performed on each sample by combining 50 µL of the sample, 55 µL isopropanol, and 20 µL of the derivation reagent, vortexing, then incubating at 60 °C for 15 min before HPLC. A total of 50 µL was injected into an isocratic mobile phase containing 5 g·L^−1^ sodium 1-heptanesulfonate monohydrate in 70:25:5 methanol:water:acetic acid flowing at 0.8 mL·min^−1^. The column and temperature conditions matched those used for ciprofloxacin HCl. The retention times of the C_1_, C_1a_, C_2a_, and C_2_ components were 2.4, 7.9, 10.3, and 11.9 min, respectively. The AUC of each component peak at 330 nm was determined and combined to determine the total drug permeated.

### 4.9. Analysis of Flux and Apparent Permeability Coefficient

Once the total amount of the drug in the receiver solution was determined, the flux was calculated as: Flux=D ÷ ΔT ÷ A where *D* is the drug permeated since the previous timepoint (nmoles), Δ*T* is the elapsed time (h) since the previous timepoint, and *A* is the permeable area (cm^2^).

The apparent permeability coefficient (P_app_, cm·h^−1^) was determined using Fick’s law: $P_{app}=\frac{Flux_{ss}}{C_{d}-C_{r}}$ where *C_d_* is the donor solution concentration (nmol·cm^−3^) (assumed constant due to excess and regular replenishment of donor solution); *C_r_* is the receiver solution concentration (considered constant at 0, since the receiver solution was replaced at each timepoint, and the results confirm that the receiver solution remained negligible); *Flux_ss_* is the average of the flux values once steady state is achieved.

### 4.10. Statistical Analysis

Statistics were performed in Prism version 9 software (GraphPad, San Diego, CA, USA). Statistical tests used for each comparison are described in each respective figure caption. A *p*-value cutoff of <0.05 was used to determine statistical significance.

## 5. Conclusions

Despite its advantages, noninvasive trans-tympanic drug delivery has seen limited use and study, likely due to the very low permeability of most drugs across the TM. Compounds, formulations, and delivery devices that may enable further development cannot be efficiently and rapidly screened in the current animal models due to the inherent costs and complexities of animal testing. This suggests that there exists a need for a method enabling the high-throughput initial screening of drug permeation across the TM.

In this study, we took a physiologically based approach at designing a simplified in vitro model that can approximate drug permeability across the TM. We determined that a model that resembles the outer epidermal layer of the TM and does not require the middle or inner TM layers is likely to represent the overall permeability of the TM. We present the drug permeability of four sample drugs in this model and determined the growth time needed for full epidermal development. There appears to be a general trend of increased drug lipophilicity leading to higher permeability, only excepted by DSP, which is likely due to its metabolism to dexamethasone rather than a true deviation from this trend. Finally, we show the effects of freeze–thaw cycles on the permeability of this model, which can be compared to our separately reported study of frozen cadaveric in situ TM.

These models seem to represent a viable and convenient tool for drug discovery advancement; however, further validation with currently accepted animal models or human tissues is needed to fully determine the accuracy of and potentially to optimize this model. The utilization of in vitro models for the initial screening of novel therapeutics continue to gain traction in drug development but unfortunately appear underutilized in otology. We believe a shift in the field that embraces these approaches would greatly benefit the millions of affected patients, clinicians, and researchers alike.

## Data Availability

Data is contained within the article and supplementary material.

## Supplementary Materials

The following supporting information can be downloaded at: https://www.mdpi.com/article/10.3390/ph15091114/s1, Figure S1: Drug Flux and TEER are Strongly Correlated; Figure S2: Fibroblast connective tissue layer has no significant effect on drug permeability in in vitro TM model; Figure S3: Permeability of ciprofloxacin in in vitro TM model matches in situ cadaveric TM permeability; Figure S4: Permeation device used for drug permeation studies and TEER measurement.
