# Supplementary material for: An In Vitro Model for Characterization of Drug Permeability across the Tympanic Membrane
Source: Pharmaceuticals (Basel). 2022 Sep 7;15(9):1114. doi: 10.3390/ph15091114 (PMC9503993; doi:10.3390/ph15091114)
Supplement: Supplementary file 1 [file pharmaceuticals-15-01114-s001.zip › pharmaceuticals-1865394-supplementary.pdf]

Supplemental Figures

An in Vitro Model for Characterization of Drug Permeability across the Tympanic Membrane

Joachim G. S. Veit [1,2], Bhaskar Birru [1,2], Ruby Singh [1,2], Elizabeth M. Arrigali [1,2] and Monica A. Serban [1,2,*]

[1] Department of Biomedical and Pharmaceutical Sciences, University of Montana, Missoula, MT 59812, USA
[2] Montana Biotechnology Center (BIOTECH), University of Montana, Missoula, MT 59812, USA
* Correspondence: monica.serban@umontana.edu

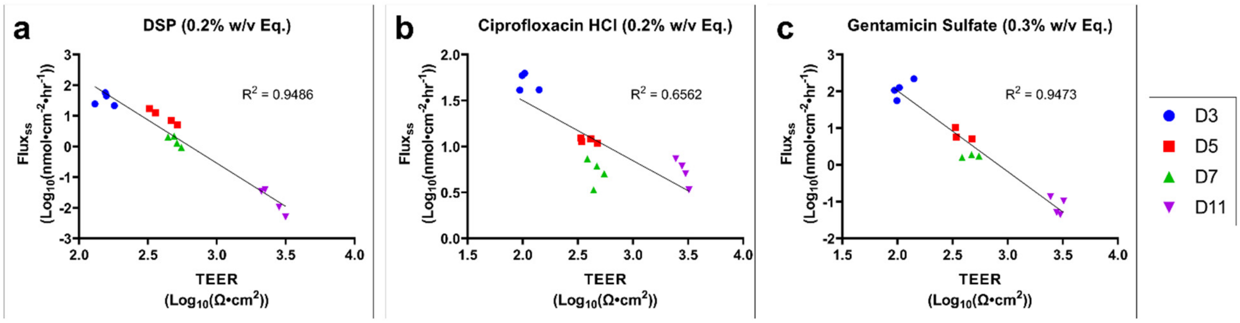

Supplemental Figure S1. Drug flux and TEER are Strongly Correlated. Correlation between steady state flux ($flux_{ss}$) and TEER after log transformation for (**a**) DSP, (**b**) ciprofloxacin HCl, and (**c**) gentamicin sulfate grown for 3, 5, 6, or 11 days. Black line shows linear regression of transformed values. n = 3-4 per condition. Legend applies to all panels. *DSP, dexamethasone sodium phosphate; Eq, molar equivalent of base compound; TEER, transepithelial electrical resistance; w/v, weight by volume.*

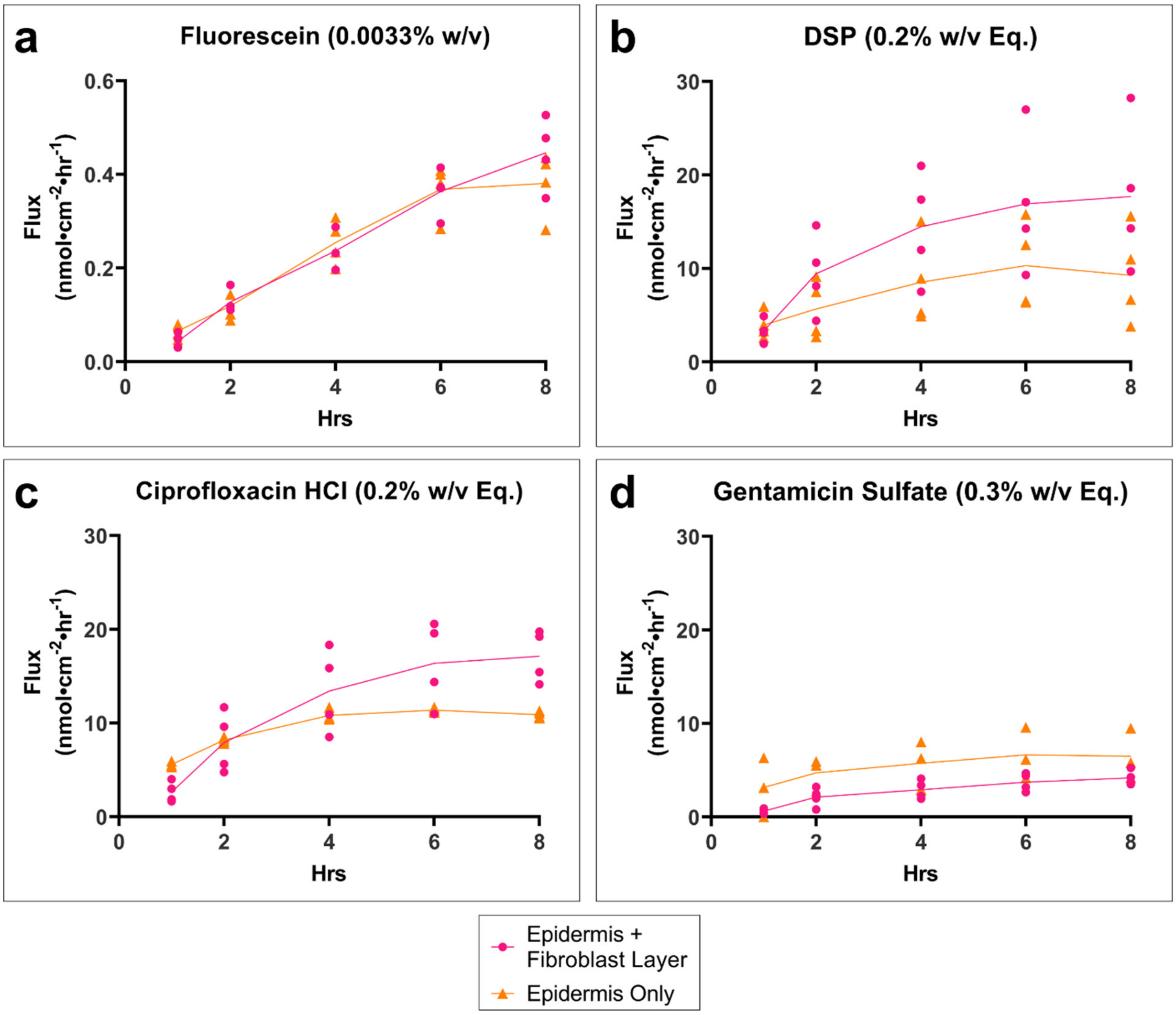

Supplemental Figure S2. Fibroblast connective tissue layer has no significant effect on drug permeability in in vitro TM model. Flux over time in TM model containing epidermis with connective tissue/fibroblasts (pink circle) or epidermis only (orange triangle) for (**a**) fluorescein, (**b**) DSP, (**c**) ciprofloxacin HCl, and (**d**) gentamicin sulfate. n = 3-4. Legend applies to all panels. *DSP, dexamethasone sodium phosphate; Eq, molar equivalent of base compound; ns, not significant;* P_{app}*, apparent permeability coefficient; TM, tympanic membrane; w/v, weight by volume.*

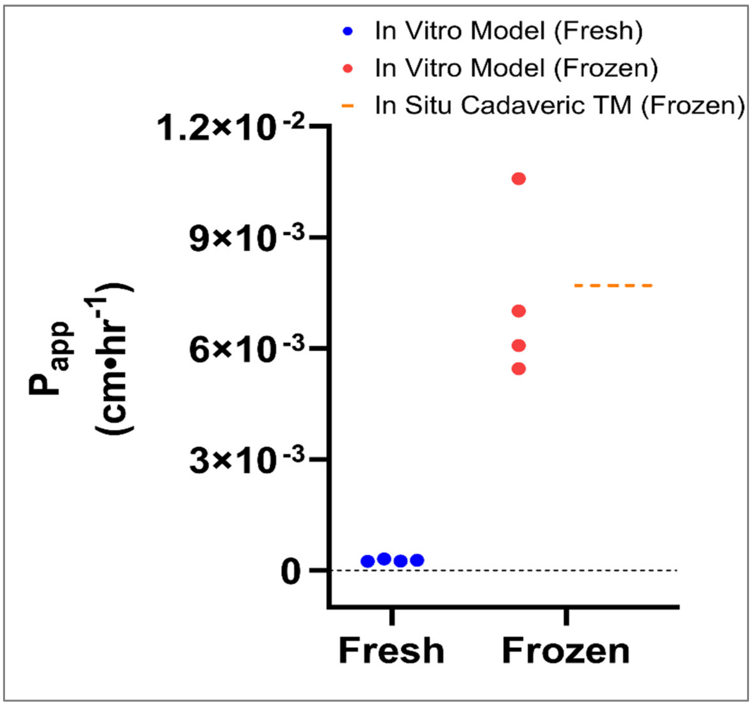

Supplemental Figure S3. Permeability of ciprofloxacin in in vitro TM model matches in situ cadaveric TM permeability. P_{app} of ciprofloxacin in in vitro TM model was compared to data from a frozen human cadaveric TM study [1]. Additionally, this fresh in vitro model data appears to align with the permeability observed in fresh in situ cadaveric TM [2]. n = 4 for in vitro data (circles); in situ data (dotted line) shows mean. *P_{app}, apparent permeability coefficient; TM, tympanic membrane.*

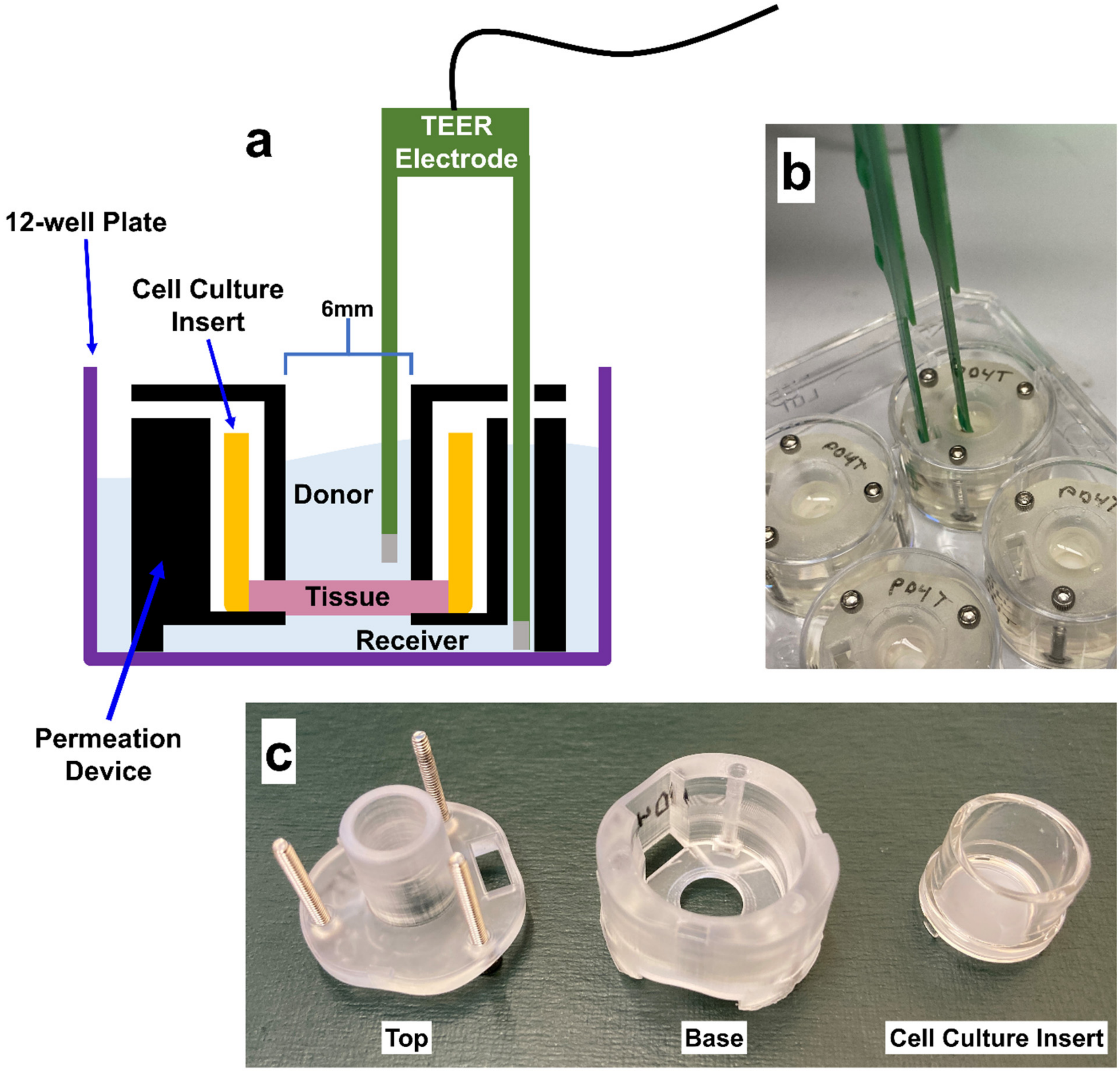

Supplemental Figure S4. Permeation device used for drug permeation studies and TEER measurement. (**a**) Cross-sectional illustration of an assembled permeation device containing a tissue on a cell culture insert during TEER testing. (**b**) Photograph of a permeation device with the TEER measurement electrode inserted to measure TEER. (**c**) Photograph of the individual parts of the permeation device. The cell culture insert is placed into the base, then the top (shown inverted) is placed over to form a seal. *TEER, transepithelial electrical resistance.*

Supplemental Information References

1. Veit, J.G.S.; Birru, B.; Wang, Y.; Singh, R.; Arrigali, E.M.; Park, R.; Miller, B.; Firpo, M.A.; Park, A.H.; Serban, M.A. An evaluation of the drug permeability properties of human cadaveric in situ tympanic and round window membranes. *Pharmaceuticals* **2022**, *15*, 1037. https://doi.org/10.3390/ph15091037.
2. Early, S.; Yang, R.; Li, X.; Zhang, Z.; van der Valk, J.C.; Ma, X.; Kohane, D.S.; Stankovic, K.M. Initial method for characterization of tympanic membrane drug permeability in human temporal bones in situ. *Front. Neurol.* **2021**, *12*, 580392. https://doi.org/10.3389/fneur.2021.580392.
